# Chair-Side Quantitative Oral-Microflora Screening for Assessing Familial Correlation of Periodontal Status and Caries Prevalence

**DOI:** 10.1371/journal.pone.0087100

**Published:** 2014-01-30

**Authors:** Yung-Kai Huang, Wei-Fang Lee, Meng-Jiy Wang, Yus-Han Sophie Chang, Wen-Shiun Tchaou, Wei-Jen Chang, Sheng-Yang Lee, Joen-Rong Sheu, Nai-Chia Teng

**Affiliations:** 1 School of Oral Hygiene, College of Oral Medicine, Taipei Medical University, Taipei, Taiwan; 2 School of Dental Technology, College of Oral Medicine, Taipei Medical University, Taipei, Taiwan; 3 Department of Chemical Engineering, National Taiwan University of Science and Technology, Taipei, Taiwan; 4 Division of Oral Rehabilitation and Center of Pediatric Dentistry, Department of Dentistry, Taipei Medical University Hospital, Taipei, Taiwan; 5 School of Dentistry, College of Oral Medicine, Taipei Medical University, Taipei, Taiwan; 6 Dental Department of Wan-Fang Hospital, Taipei Medical University, Taipei, Taiwan; 7 Department of Pharmacology, School of Medicine, College of Medicine, Taipei Medical University, Taipei, Taiwan; 8 Graduate Institute of Medical Sciences, College of Medicine, Taipei Medical University, Taipei, Taiwan; University of Toronto, Canada

## Abstract

Aim: Our goal was to investigate the relationship between clinical status and the presence of carious or periodontal pathogens among parent-child familial pairs. Clinical practices of risk assessment with consideration of familial pathogen interaction might reduce the need for therapy, improve patient outcomes, and ultimately reduce oral disease burden. Materials and Methods: In this study, we enrolled 30 parent-child pairs, with the children exhibiting complete deciduous dentition or mixed dentition with only permanent first molars. Clinical statuses were evaluated using caries and periodontal disease indicators, including the sum of decay and the number of missing or filled teeth (DMFT) for adults, decay, extraction caused by dental disease, and filled teeth (deft), for children, probing depth, and plaque control record (PCR). Supra- and sub-gingival bacteria were determined based on semi-quantitative measurements of microbial infection by using data from the Dentocult^®^ SM test (caries-related organisms) and the PerioCheck^®^ test (periodontal disease-related organisms). Results: No statistically significant relationship was detected between the prevalence of periodontal pathogens and that of cariogenic pathogens in the oral cavity. However, the clinical status of caries (DMFT) was negatively correlated with the clinical status of periodontal disease (pocket depth) in parents who were infected with dominant periodontal pathogens (*r = *−0.59, *p*<0.01). Parents’ DMFT scores were positively correlated with children’s deft and PCR scores. PCR and deft scores of children appeared to decrease significantly with the parent’s pocket depth. Conclusion: The study showed that the quantity of caries pathogens were not significant related to periodontal pathogens, but the caries clinical outcome is negative related with periodontal clinical outcome between familial pairs.

## Introduction

Dental caries and periodontitis are the most widespread oral diseases in populations of all ages. Recent epidemiological surveys have revealed that 60% of the U.S. population present with mild forms of periodontal disease and that 64% of seniors (adults aged 65 y and older) exhibit either moderate or severe periodontitis [1]. Various factors including nutritional status, tobacco and alcohol use, hygiene, and stress have been linked to a wide range of oral diseases, forming the foundation of the commonly used risk-factor approach for preventing oral diseases [2], [3]. Among these, oral hygiene is one of the most critical factors for preventing oral diseases, particularly caries and periodontal disease [4]. The relationship between oral hygiene and intra-oral pathogenesis has been defined, and the advanced relationship between pathogenesis and dental caries or periodontal disease has also been investigated [4], [5]. The core of traditional dentistry is based on the treatment of oral diseases. For dental caries, treatment is typically initiated when lesions are clinically detectable and tissue damage is irreversible; a similar approach can be applied in treating periodontal disease. Remarkable advances have now been made in the field of oral microbiology, particularly with regards to adjunct diagnosis.

*Streptococcus mutans* is the principal cariogenic pathogen [6], [7]. The early acquisition of *S. mutans* has been reported to be due to mother-to-child transmission: children whose mothers have high levels of salivary *S. mutans* acquire caries earlier and also have higher counts of *S. mutans* than have children whose mothers have low levels of *S. mutans* [8]. *Actinobacillus actinomycetemcomitans*, *Porphyromonas gingivalis*, *Prevotella intermedius*, and *Bacterioids forsythus* are the strongest putative pathogens in periodontitis [9]. Moreover, the occurrence of intrafamilial transmission of periodontal pathogens has been suggested by previous studies. Parents are the first people who interact with their children and are thus a potential source of infection, and the detection of periodontal pathogens in children at an early age may indicate endogenous infection [10], [11].

People with salivary bacterial counts of *S. mutans* and *P. gingivalis* in excess of 1×10^5^, and 4×10^6^ colony forming units (CFU)/mL are regarded to have a high risk of developing dental caries and periodontitis, respectively. However, little research supports a relationship between streptococci (*S. mitis* and *S. sanguis*) and periodontal pathogens. Previous in vitro studies have suggested that the growth of *S. mutans* is related to *P. gingivalis* [12], [13], but no direct evidence has been presented of this relationship in the human oral cavity. Microbial tests, such as Dentocult® SM Strip and PerioCheck®, have been developed that are easy to use at the chair-side. The Dentocult test is a simple and clinically acceptable method that might facilitate the procedure of determining the amount of bacteria present in dental plaques and mobilized into the saliva. PerioCheck® is a rapid chair-side test used to detect the presence of neutral proteases [14]. The presence of neutral proteases has been implicated in collagen breakdown, which is a key feature of periodontal disease [15]. The PerioCheck® method has been demonstrated to exhibit a high sensitivity (88%) when diagnosing periodontitis [16]. These diagnostic tests can detect the presence of active disease, predict future disease progression, and evaluate the response to clinical therapy, thereby improving the clinical management of patients. However, thus far, a comprehensive understanding of the pathogenesis of dental caries and periodontal disease, particularly the transmission between child and parent or caregiver, has been lacking. In this study, we investigated the relationship between clinical status and the presence of carious or periodontal pathogens among child-parent pairs. Clinical practices of risk assessment with a consideration of familial pathogen interaction might reduce the need for therapy, improve patient outcomes, and ultimately reduce the burden of oral disease.

## Materials and Methods

### Participants

Thirty parent-child pairs were recruited for this study. Children aged 3–7 years exhibiting complete deciduous dentition or mixed dentition with only permanent first molars were considered eligible for enrollment. The parents of all enrolled children had no chronic or systemic diseases, were not pregnant, and had no history of dental disease beside caries. The study was approved by the Taipei Medical University Joint Institutional Review Board. Before conducting interviews and collecting specimens, written informed consent was obtained from the parents of all children who participated in the study. The study complied with the World Medical Association Declaration of Helsinki.

### Caries Examination and Oral Hygiene Evaluation

A questionnaire was completed by parents to provide information on their socioeconomic status and their child’s oral health habits (infant feeding history, current diet, oral hygiene practices) and systemic health and medication. All children were examined by a dentist who was trained and assessed in relation to an experienced oral epidemiologist. The dentist demonstrated >90% reliability before the training session was completed. Dental examination for caries was conducted in fully equipped dental units, using an illuminated mouth mirror and a CPI probe (CP-11.5B6, Hu-Friedy, Chicago, IL, US). The tooth status was assessed through visual inspection, aided by tactile inspection if necessary. The World Health Organization (WHO) criteria for the diagnosing and coding of dental caries were used. Dental caries were scored using the sum of decay and the number of missing or filled teeth (DMFT) for adults and decay, extraction caused by dental disease, and filled teeth (deft) for children. The plaque control record (PCR) developed by O’Leary was used to examine the dental hygiene status of parents and children [17].

### Periodontal Status and Classification

To evaluate periodontal health, periodontal examinations were performed on adults, which included full mouth periodontal charting that was conducted using a periodontal probe and the gingival index. The American Dental Association periodontal classification was used to identify or categorize each patient’s periodontal status. After clinical and radiographic data were gathered, the patients were classified into one of the 4 case types: Type I, for gingivitis; Type II, for early periodontitis; Type III, for moderate periodontitis; and Type IV, for advanced periodontitis. A similar assessment was not performed on the children because most children do not exhibit severe periodontal disease.

### Oral Bacteria Tests

Study participants were asked to refrain from using oral hygiene procedures the night before the day of the study. Saliva collection was scheduled at the beginning of the day. Participants were first asked to swallow preexisting saliva. Subsequently they were to chew a standard piece of paraffin wax (Orion Diagnostics kit). The saliva that was produced was collected in a sterile plastic recipient. A minimum of 2 mL of saliva had to be collected. The plaque sample was collected from every quadrant by using sterile mini-brushes. The collection was from the interproximal area between the primary first molar and primary second molar. Immediately after collection, both the saliva and the plaque *S. mutans* samples were assessed using the Dentocult SM® dip strip (Orion Diagnostica, Espoo, Finland). The growth density on the strips was classified according to the manufacturer’s instructions and expressed as scores of 0, 1, 2, and 3, which correspond to *S. mutans* growth levels of ≤104, ≤10^5^, 10^5^–10^6^, and ≥10^6^ CFU/mL.

PerioCheck® (CollaGenex Pharmaceuticals, Newtown, PA, USA) is a quick and simple periodontal diagnostic tool that is used to detect peptidase activity specifically attributed to *Treponema denticola*, *P. gingivalis*, and *B. forsythus*. Periodontal pathogens residing in the periodontal pocket react specifically to the peptidase. A composite score ranging from 0 to 2, assigned based on comparing the blue color of the test result with a standard color chart, was used to determine the amount of bacteria in samples.

### Statistical Analyses

Data were analyzed using SAS 9.2 software (Cary, NC, USA). Student’s *t* test was used to test for significant differences in DMFT, deft or PCR scores of samples in relation to demography strata, oral hygiene strata, or a pathogen level of 0 or above. Analysis of variance (ANOVA) was used to test the PCR and deft scores of children among the children’s age groups and main caregiver categories. Fisher's exact test was used to evaluate differences in the categorical variables of periodontal status and caries indices. The strength of the association between periodontal indices (PCR, gingival index, and depth) and the caries index (DMFT) was noted using a correlation coefficient. Linear regression was used to elucidate the relationship between parent DMFT and child deft or PCR scores. A probability level (alpha) of less than 0.05 was used as the criterion for significance.

## Results

Table 1 presents the PCR and deft scores in relation to demography and oral hygiene habits of children. The deft scores of children who routinely ate or drank before bedtime were significantly higher than those of children who did not have the habit of eating or drinking before bedtime. The PCR scores of children who brushed their teeth less frequently than daily were significantly higher than those of children whose brushing frequency was once or more per day. The PCR scores of children whose caregivers’ occupation were professional employee were significantly lower than those of children whose caregivers’ occupation were other three categories.

**Table 1:** Mean PCR and deft scores of children according to demography and oral hygiene habits in children and caregivers.

		PCR	p- value	deft	p- value
	N	Mean ± SE		Mean ± SE	
Gender			0.08^a^		0.39^a^
Girls	19	78.35±7.38		9.47±1.04	
Boys	11	92.40±2.47		7.82±1.76	
Age (y)			0.10^b^		0.81^b^
3–4	12	86.80±4.45		9.58±1.45	
5	10	69.76±12.80		8.20±1.65	
6–7	8	95.73±1.39		8.63±1.37	
Single child			0.08^a^		0.28^a^
No	21	89.08±4.37		9.52±1.06	
Yes	9	70.50±12.06		7.33±1.80	
Eat/drink before bedtime			0.08^a^		<0.01^a^
No	18	77.70±7.79		6.44±1.01	
Yes	12	92.20±2.12		12.50±1.08	
Snack consumption			0.44^a^		0.73^a^
Rare	7	73.19±15.87		8.29±2.49	
Yes	23	86.64±4.25		9.04±0.96	
Frequency of brushing			<0.01^a^		0.54^a^
Less than daily	2	100.00±0.00		11.00±1.00	
Once or more per day	28	82.32±5.16		8.71±0.98	
Floss used			0.30^a^		0.44^a^
No	18	87.69±5.46		9.00±1.19	
Yes	12	77.22±9.03		8.67±1.49	
Main caregiver			0.59^b^		0.44^b^
Mother	15	80.61±9.89		9.60±0.89	
Mother and father	9	81.55±9.48		9.22±2.90	
Others	6	93.65±3.37		6.50±2.20	
Main caregiver’s education			0.09^a^		0.40^a^
High school	7	92.85±3.60		10.29±1.04	
University and above	23	80.66±6.18		8.43±1.15	
Main Caregiver’s occupation			0.02^b^		0.36^b^
Employer/professional	6	94.13±2.59		11.67±1.2	
Employee/professional	8	59.67±14.56		6.75±2.42	
Employee/non-professional	10	87.97±4.64		9.00±1.53	
Unemployed	6	97.22±1.96		8.67±1.48	
Floss used frequency of main caregiver			0.48^b^		0.73^b^
Occasion	18	88.31±5.20		9.39±1.20	
Once/day	7	75.07±12.07		8.57±1.76	
Twice/day	5	78.00±6.17		7.40±2.71	
Dental care utilization of main caregiver			0.78^b^		0.70^b^
Once/half year	4	92.2±4.34		8.25±2.46	
Once/one year	14	83.07±8.1		9.71±1.52	
Once/more than one year	12	81.11±7.85		8.08±1.29	

a p value for student t test.

b p value for ANOVA test.

Abbreviations: deft, decay, extraction, and filled teeth; PCR, plaque control records; SE, standard error.

The results in Table 2 reveal the association between the use of the chair-side semi-quantitative assays to measure microbial infection and the caries index or periodontal clinical status in the parent and child. The mean scores of DMFT and deft were 12.90 and 8.87 in parents and children, respectively. The mean DMFT score of parents who tested positive for plaque *S. mutans* was higher than that of parents who tested negative (p = 0.03, student *t*-test). Parents identified to carry microbial infections (based on the *S. mutans* Dentocult SM® and PerioCheck® results) had a significantly higher DMFT score than parents who tested negative for microbial infection. Periodontal clinical status was only assessed on parents and the mean periodontal pocket depth and gingival index were 2.11 mm and 1.37, respectively. Plaque *S. mutans*-positive parents had a higher gingival index than parents who tested negative for plaque *S. mutans* (p = 0.04). Parents who tested positive on the PerioCheck® test had higher a gingival index than those who tested negative (p = 0.04).

**Table 2:** Association between the results of chair-side semi-quantitative microbial infection measurements and caries index or periodontal clinical status in parent and child.

Microbial results - parents		DMFT	PCR	Pocket depth	Gingival index
	N	Mean ± SE			
Plaque SM					
0	8	10.13±1.93	75.02±7.65	2.22±0.25	1.26±0.08
1–3	22	13.91±0.85	86.65±2.86	2.07±0.15	1.61±0.09
p value^a^		0.04	0.18	0.62	0.04
Saliva SM					
0	6	9.33±2.41	71.89±10.05	2.37±0.32	1.37±0.13
1–3	24	13.79±0.81	86.46±2.63	2.05±0.13	1.55±0.09
p value^a^		0.03	0.05	0.31	0.36
PerioCheck®					
0	8	10.00±1.33	82.68±5.37	1.92±0.23	1.26±0.09
1–2	22	13.95±0.93	83.86±3.67	2.18±0.15	1.61±0.09
p value^a^		0.04	0.86	0.37	0.04
**Microbial results - children**	**N**	**deft**	**PCR**		
Plaque SM					
0	9	7.44±2.14	66.45±13.79		
1–3	21	9.48±0.95	90.81±2.78		
p value^a^		0.31	0.11		
Saliva SM					
0	13	8.08±1.61	75.93±10.24		
1–3	17	9.47±1.07	89.29±3.32		
p value^a^		0.46	0.23		
PerioCheck®					
0	9	7.66±1.38	84.77±9.57		
1–2	21	9.38±1.17	82.96±5.78		
p value^a^		0.40	0.73		

a p value for student t test.

Abbreviations: DMFT, decay and the number of missing or filled teeth; deft, decay, extraction, and filled teeth; PCR, plaque control records; SE, standard error; SM, *Streptococcus mutans*.

The results in Table 3 indicate the association between periodontal and cariogenic pathogens. Relative to parents who tested negative for plaque *S. mutans*, the parents who tested positive showed higher rates of periodontal pathogens, but the difference was not statistically significant (50.00% vs. 81.82%, p = 0.16). The parents’ status of periodontal pathogens (determined using PerioCheck®) was not associated with the children’s *S. mutans* or pathogen status. These results suggest that no statistically significant relationship exists between the prevalence of periodontal pathogens and that of cariogenic pathogens in the oral cavity.

**Table 3:** Association between periodontal and cariogenic pathogens.

	Parent PerioCheck®			Child PerioCheck®	
	0	1+		0	1+
	N (%)			N (%)	
Parent plaque SM			Child plaque SM		
0	4 (50.00)	4 (18.18)	0	4 (44.44)	5 (23.81)
1+	4 (50.00)	18 (81.82)	1+	5 (55.56)	16 (76.19)
p value^a^	0.16		p value^a^	0.39	
Parent saliva SM			Child saliva SM		
0	3 (37.50)	3 (13.64)	0	6 (66.67)	7 (33.33)
1+	5 (62.50)	19 (86.36)	1+	3 (33.33)	14 (66.67)
p value^a^	0.30		p value^a^	0.12	
Child plaque SM			Parent plaque SM		
0	4 (50.00)	5 (22.73)	0	2 (22.22)	6 (28.57)
1+	4 (50.00)	17 (77.27)	1+	7 (77.78)	15 (71.43)
p value^a^	0.20		p value^a^	1.00	
Child saliva SM			Parent saliva SM		
0	5 (62.50)	8 (36.36)	0	2 (33.33)	7 (29.17)
1+	3 (37.50)	14 (63.64)	1+	4 (66.67)	17 (70.83)
p value^a^	0.24		p value^a^	1.00	
Child PerioCheck®			Parent PerioCheck®		
0	2 (22.22)	6 (28.57)	0	4 (50.00)	5 (22.73)
1+	7 (77.78)	15 (71.43)	1+	4 (50.00)	17 (77.27)
p value^a^	1.00		p value^a^	0.20	

a p value for Fishers exact test.

SM: *Streptococcus mutans.*

**Figure 1:**
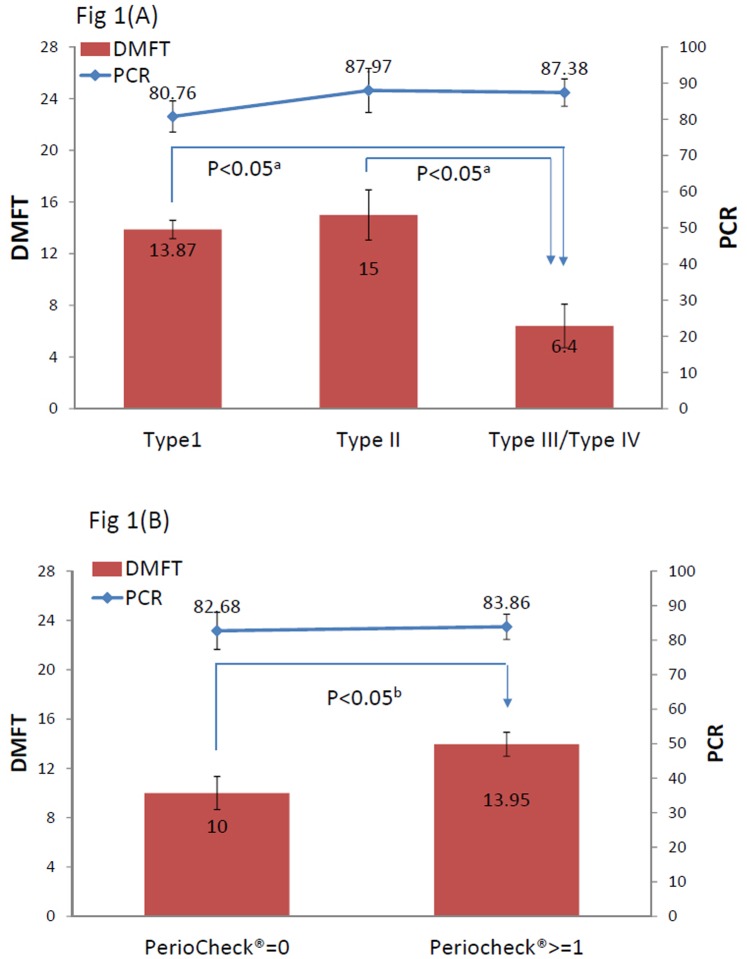
Comparison of DMFT and PCR scores in relation to periodontal status in parents. (A): Distribution of DMFT and PCR scores among periodontal classification types. (B): DMFT and PCR score distribution in relation to PerioCheck® status. ^a^Significantly different (p<0.05) from periodontal classification Type I based on analysis of variance and Scheffe’s test. ^b^Significantly different (p<0.05) based on Student’s *t* test. Abbreviations: DMFT, decay and the number of missing or filled teeth; PCR, plaque control records.

To explore the relationship between the periodontal clinical status or periodontal pathogens and caries indices, we compared the DMFT and PCR scores and the periodontal status in parents (Fig. 1). The DMFT scores of parents who exhibited moderate periodontitis (Type III) or advanced periodontitis (Type IV) were significantly lower than those of parents who had gingivitis (Type I) or early periodontitis (Type II) (Fig. 1A). The DMFT scores of patients without any detectable dominant periodontal pathogens (PerioCheck® = 0) were lower than those of patients in whom the dominant periodontal pathogens were present (PerioCheck® ≥ 0). The rates of testing negative for periodontal pathogens (PerioCheck® = 0) in the periodontal classification Types I, II, and III/IV were 75%, 12.5%, and 12.5%, respectively, and the PerioCheck® test results were not significantly associated with the periodontal classifications (p = 0.58 in the χ^2^ test). By contrast, the status of cariogenic pathogens (plaque or saliva *S. mutans*) was associated with DMFT scores, but not with pocket depth (data not shown).

Table 4 presents the correlation coefficients for caries indices and periodontal status in parents. The DMFT score was negatively correlated with pocket depth (*r* = −0.49, *P*<0.01), but the correlation was not observed in patients in whom the dominant periodontal pathogens were not detected (PerioCheck® = 0). The negative correlation was stronger (*r* = −0.59, *P*<0.01) in patients with dominant periodontal pathogens when compared with all patients. These results indicate that DMFT scores and pocket depth are correlated in an oral environment containing periodontal dominant pathogens.

**Table 4:** Correlation coefficients of caries indices and periodontal status in parent.

	PCR	Gingival index	Pocket depth
DMFT	−0.02	0.20	−0.49**
PCR	1.00	0.24	0.19
Gingival index		1.00	0.15
Pocket depth			1.00
**PerioCheck® = 0, N = 8**			
	**PCR**	**Gingival index**	**Pocket depth**
DMFT	0.45	−0.14	−0.63
PCR	1.00	0.24	0.19
Gingival index		1.00	0.15
Pocket depth			1.00
**PerioCheck® ≥ 1, N = 22**			
	**PCR**	**Gingival index**	**Pocket depth**
DMFT	−0.16	0.11	−0.59**
PCR	1.00	0.34	0.25
Gingival index		1.00	0.15
Pocket depth			1.00

Abbreviations: DMFT, decay and the number of missing or filled teeth; PCR, plaque control records.

** p<0.01.

**Figure 2:**
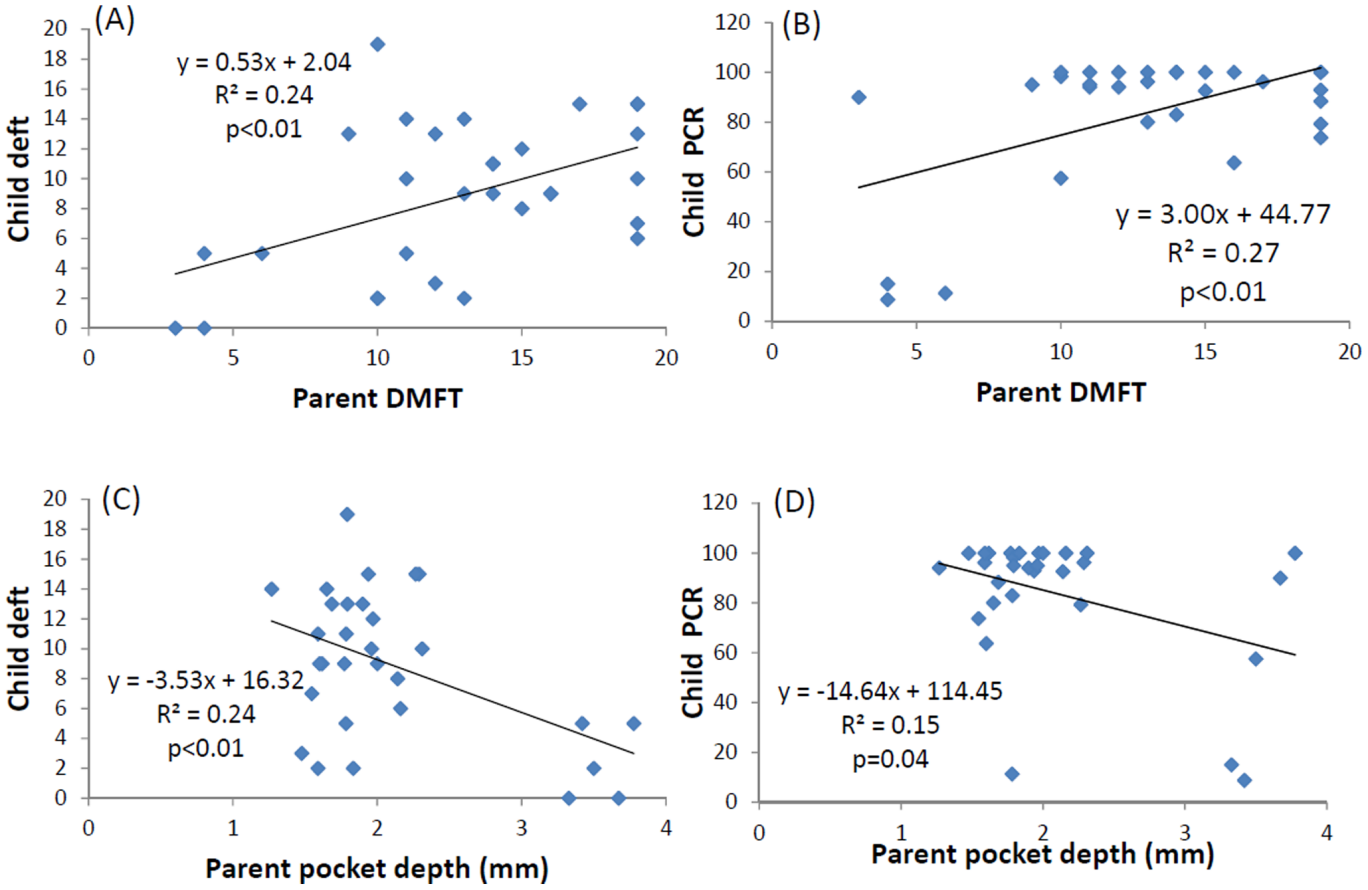
Scatterplots indicating the relationships between child caries index and parent caries index or clinical periodontal outcome. Scatterplots of (A) child deft and parent DMFT scores; (B) child PCR and parent DMFT scores; (C) child deft scores and parent pocket depth; and (D) child PCR scores and parent pocket depth. Abbreviations: deft, decay, extraction, and filled teeth; DMFT, decay and the number of missing or filled teeth; PCR, plaque control records.

We examined the relationship between the dental caries index of children (PCR and deft) and the parents’ caries clinical status or periodontal clinical status (Fig. 2). The PCR and deft scores of children appeared to increase significantly with the parents’ DMFT scores increasing (Figs. 2A and 2B) and appeared to decrease with the parent’s pocket depth increasing (Figs. 2C and 2D).

## Discussion

The results in Table 1 indicate that the children’s deft score was influenced by the habit of eating or drinking before bedtime. The children’s PCR score was influenced by the frequency of brushing. We used the habit of eating or drinking before bedtime as a factor in a multivariate model in which children’s deft score was used as a dependent variable. The association between children’s deft score and parents’ DMFT score or parents’ pocket depth was statistically significant (β = 0.45, p<0.01 for parents’ DMFT; β = −0.38, p<0.01 for parents’ pocket depth). We used the frequency of brushing as a factor in a multivariate model in which children’s PCR score was used as a dependent variable. The association between children’s PCR score and parents’ DMFT score or parents’ pocket depth was statistically significant (β = 2.94, p<0.01 for parents’ DMFT; β = −14.4, p = 0.03 for parents’ pocket depth).

Dental caries and periodontal disease are the most common diseases of the oral cavity. Both diseases are known to be associated with microorganisms that colonize the tooth surface. The influence of the family in the establishment of pathogenic microorganisms in children has been documented [18], [19]. However, even when an oral disease is present, a microbial sample is rarely taken and tested to confirm the presence of microorganisms [20]. Because dental caries and periodontal disease have been reported to have a genetic basis [21], certain people may be predisposed to or have a greater likelihood of hosting these bacteria and might thus suffer from poor oral health. Knowledge of the underlying familial microbial infection could be used in assessing the risk of disease development and progression, which would facilitate dental counseling and the use of preventive protocols.

It is very interesting that the positive detection of *S. mutans* in parents by using the Dentocult® SM strip test was highly correlated with positive detection in their children. When parents exhibited *S. mutans*-positive plaques, their children carried the plaque *S. mutans* at a rate that was significantly higher than in children of parents who tested negative for plaque *S. mutans* (90.48% vs. 33.33%). A strong association was also detected between the presence of plaque *S. mutans* in parents and the presence of saliva *S. mutans* in their children (data not show). Microbial monitoring has been considered as an alternative method for evaluating current caries activity and future caries risk. Chair-side evaluation of salivary and plaque *S. mutans* levels (e.g., Dentocult-SM) has been used in numerous studies [22]. The practicality of the test in children has been evaluated and the test has been demonstrated to be accurate [23]. Moreover, the microbial counts on the strip have been determined to be an accurate indicator of infection [24]. A systematic review has confirmed that the presence of *S. mutans* in both the plaque and the saliva of young caries-free children was associated with a considerable increase in the risk of early childhood caries (ECC) [25].

In this study, we determined that the chair-side Dentocult® SM score may be an indicator of the present clinical status only for parents (Table 2): the Dentocult SM score was not significantly related to deft status. The paired child-deft and parent-DMFT scores were positive correlated (Fig. 2A), indicating that genetic factors might play a key role in the clinical status of dental caries. The levels of *S. mutans* in saliva and plaque were shown to predict caries activity, but the clinical status of the children was more closely related to their parents’ clinical status in this cross-sectional study.

In this study, the parents’ scores on both the Periocheck® and the Dentocult SM tests were correlated with the gingival index (Table 2). The presence of high numbers of pathogens at the site of the tooth may induce gingival inflammation, but the quantity of pathogen was related neither to the pocket depth nor to the PCR. A previous cross-sectional analysis indicated that *P. gingivalis* from tongue and subgingival samples and *B. forsythus* from subgingival samples were associated with early periodontitis in adults [26]. Watson et al. (1991) reported that young children are colonized by *P. gingivalis*, *T. denticola*, and *T. forsythensis*. Children whose parents were colonized by bacteria that test positively with benzoyl-DL-arginine-naphthylamide (BANA) were significantly more likely to exhibit a positive BANA reaction than children of parents who were not colonized by BANA test-positive bacteria. Furthermore, children displaying BANA-positive plaques were more likely than children without such plaques to have parents, caregivers, or other family members who were older and had a history of periodontal disease [27], [28]. In this study, we noted that parents who were PerioCheck® positive were more likely have children who tested positive in PerioCheck® than parents who were PerioCheck® negative, but the difference was not statistically significant (Table 4). Our results agree with the previous studies in which the BANA test was used.

Our results indicated that the PerioCheck® score was not correlated with saliva *S. mutans* (Table 3). Moreover, the DMFT scores of patients with moderate or advanced periodontitis were lower than those of gingivitis patients (Fig. 1). Based on the results, we conclude that plaque *S. mutans* and PerioCheck® scores were correlated with gingival status. These data suggest that PerioCheck® can serve as a chair-side indicator of clinical severity, but gingivitis is more related to supragingival pathogens. Recently, periodontal therapy has been reported to lead to a change in microbial flora, from a predominantly periodontal pathogenic organism to one that is more cariogenic [29], [30]. Patients whose periodontal disease had been treated were found to exhibit high microorganism recovery during follow-up [31], [32], and, similarly, patients who had been treated for periodontal disease showed higher *S. mutans* levels than untreated patients did [29]. Periodontitis patients who were not treated displayed high recovery rates of *S. mutans* in saliva, tongue dorsum, buccal mucosa, and supra- and sub-gingival plaque [33].

The salivary levels of *P. gingivalis* and *S. mutans* have been assessed using the polymerase chain reaction method to compare periodontal disease and dental caries [34]. The results suggested that the salivary levels of *S. mutans* were significantly higher in the healthy group than in periodontitis patients, and that salivary levels of *P. gingivalis* were significantly higher in the caries-free group than in the periodontally healthy group with caries. Data obtained using semi-quantitative detection tools in this study did not reveal an inverse correlation between *S. mutans* and periodontic pathogens. Furthermore, patients exhibiting poor oral hygiene had both higher saliva *S. mutans* levels and Periocheck® scores, both in parent and child groups (Fig. 2).

Although antagonistic interactions between *S. sanguis* and *Porphyromonas* species in vivo have been reported, these interactions might be related to the resistance to colonization after infection with gram-positive streptococci [13], [35]. In these studies, polymerase chain reaction was used to detect the pathogens, and some of the samples were collected from gingival sulcus. Despite the changes in the proportion of *S. mutans* in the subgingival sample before and after therapy, the bacterium persisted in the oral cavity of patients treated for periodontitis. The subgingival area is an ecological niche, and the high proportions of *S. mutans* in this niche after periodontal therapy indicate a shift from gram-negative bacteria to gram-positive bacteria including *S. mutans*. In this study, we did not use polymerase chain reaction to detect oral pathogens, but instead used a method that is more practical for the clinician. Dentocult SM was used to detect the presence of *S. mutans* in supragingival plaques and saliva, and PerioCheck® was used to detect the organism in the subgingival plaque. The periodontal and caries pathogens, which resided in the oral cavity but not in the same ecological niche, showed no antagonistic interactions.

In this study, we did not detect an inverse correlation between the scores of PerioCheck® and DMFT, but parents with severe periodontitis had significantly lower DMFT scores (Fig. 1A), which suggests high rates of familial transmission and habitation of the pathogens. Because the periodontitis status of children cannot be detected, PerioCheck® serves as a crucial indicator of the early acquisition of these organisms by children. The periodontitis risk-assessment model should include a pathogen score and the clinical status (DMFT and periodontal status) of the caregiver or parent; the use of such a model could help identify children who face a high risk of periodontal disease and exclude those whose risk of developing the disease is low. An understanding of the risk factors involved in initial periodontitis and the use of an appropriate diagnostic sampling protocol will be invaluable not only in identifying people who face the risk of suffering from periodontitis, but also in developing effective disease intervention measures [36].

Another key result obtained in this study was the correlation detected between the pocket depth and the caries index. Our data indicate that parents exhibiting greater pocket depth developed fewer dental caries (Table 4), and, moreover, that their children had fewer dental caries (Fig. 2C). The clinical severity of the periodontal status appeared to be inversely correlated with dental caries, particularly when the pathogen could be isolated. Therefore, both pathogen detection and genetic factors should be considered in risk assessment when evaluating periodontal and dental caries clinical status in children.

This cross-sectional study was conducted to elucidate the familial correlation of microbial levels with periodontal status and caries development. The 30 parent-child pairs were recruited from dental clinics, and the caries- and periodontal-related microbial levels were measured using semi-quantitative microbial commercial kits. This study should be extended using a larger sample size.

People who are at risk of suffering from periodontal disease or dental caries could be identified and targeted for intervention. Moreover, understanding the interaction between genetic and environmental factors is critical: evidence indicates that dental caries and periodontitis are multifactorial infectious diseases caused by numerous contributory environmental factors and that a strong genetic component is involved in the etiology of these diseases. Additional studies on twins, families, and animal models should be conducted to broaden the current understanding of the genetic component of dental caries and periodontitis.
